# Ultra-low dose naltrexone attenuates chronic morphine-induced gliosis in rats

**DOI:** 10.1186/1744-8069-6-22

**Published:** 2010-04-16

**Authors:** Theresa-Alexandra M Mattioli, Brian Milne, Catherine M Cahill

**Affiliations:** 1 Department of Pharmacology & Toxicology, Queen's University, Kingston, Ontario, K7L 3N6, Canada; 2 Centre for Neuroscience Studies, Queen's University, Kingston, Ontario, K7L 3N6, Canada; 3 Department of Anaesthesiology, Kingston General Hospital, Queen's University, Kingston, Ontario, K7L 2V7, Canada

## Abstract

**Background:**

The development of analgesic tolerance following chronic morphine administration can be a significant clinical problem. Preclinical studies demonstrate that chronic morphine administration induces spinal gliosis and that inhibition of gliosis prevents the development of analgesic tolerance to opioids. Many studies have also demonstrated that ultra-low doses of naltrexone inhibit the development of spinal morphine antinociceptive tolerance and clinical studies demonstrate that it has opioid sparing effects. In this study we demonstrate that ultra-low dose naltrexone attenuates glial activation, which may contribute to its effects on attenuating tolerance.

**Results:**

Spinal cord sections from rats administered chronic morphine showed significantly increased immuno-labelling of astrocytes and microglia compared to saline controls, consistent with activation. 3-D images of astrocytes from animals administered chronic morphine had significantly larger volumes compared to saline controls. Co-injection of ultra-low dose naltrexone attenuated this increase in volume, but the mean volume differed from saline-treated and naltrexone-treated controls. Astrocyte and microglial immuno-labelling was attenuated in rats co-administered ultra-low dose naltrexone compared to morphine-treated rats and did not differ from controls. Glial activation, as characterized by immunohistochemical labelling and cell size, was positively correlated with the extent of tolerance developed. Morphine-induced glial activation was not due to cell proliferation as there was no difference observed in the total number of glial cells following chronic morphine treatment compared to controls. Furthermore, using 5-bromo-2-deoxyuridine, no increase in spinal cord cell proliferation was observed following chronic morphine administration.

**Conclusion:**

Taken together, we demonstrate a positive correlation between the prevention of analgesic tolerance and the inhibition of spinal gliosis by treatment with ultra-low dose naltrexone. This research provides further validation for using ultra-low dose opioid receptor antagonists in the treatment of various pain syndromes.

## Background

Opioid drugs, such as morphine, are widely used for the management of moderate to severe pain. Unfortunately, the usefulness of morphine and other opioid analgesics in the management of pain is limited due to the development of tolerance to the analgesic effects of these drugs with repeated exposure [1]. Clinically, the onset of tolerance necessitates increasing doses of opioids, which in turn typically increases the number and severity of adverse effects and compliance [2].

Morphine acts to inhibit nociception predominately via G_i _protein-coupled μ-opioid receptors [3,4] located in nociceptive pathways throughout the central nervous system including the dorsal spinal cord. Within the spinal cord, μ-opioid receptors are well recognized to localize on pre- and post-synaptic nociceptive neurons, but they are also present on astrocytes and microglia [5-10], however the function of μ-opioid receptors on glial cells remains elusive.

A number of factors appear to contribute to the development of analgesic tolerance. In general, the development of tolerance is thought to involve cellular adaptation/modulation that results in decreased analgesic potency. The precise mechanism(s) of action is not known; however, investigators have been able to attenuate or reverse established analgesic tolerance to morphine by inhibiting either the release of neurotransmitters and/or inhibition of their receptors [11-16]. Within the last decade, activation of spinal glia has emerged as a novel mechanism underlying analgesic tolerance [17-19]. Relevant to the current study, the administration of sub-therapeutic (ultra-low) doses of opioid specific antagonists (e.g. naloxone, naltrexone) augmented opioid-induced analgesia and inhibited and/or reversed the development of tolerance and physical dependence [20]. Although this relationship was studied intensively in various *in vitro *and *in vivo *models [20-22], only recently have clinical trials been undertaken to investigate the improved therapeutic benefit of combining opioid analgesics with ultra-low dose opioid receptor antagonists. To date, clinical trials have confirmed that combinations of opioids and ultra-low dose antagonists both enhance and prolong opioid-induced analgesia, and prevent analgesic tolerance and physical dependence [23,24]. Precisely how ultra-low dose antagonists prevent/reverse tolerance to opioid analgesics is not fully understood, but spinal glia may play a crucial role. We demonstrate that one contributing mechanism is that ultra-low dose naltrexone blocks opioid-induced activation of spinal glial cells.

## Results

### Ultra-low dose naltrexone attenuated the development of tolerance to morphine antinociception

**Figure 1:**
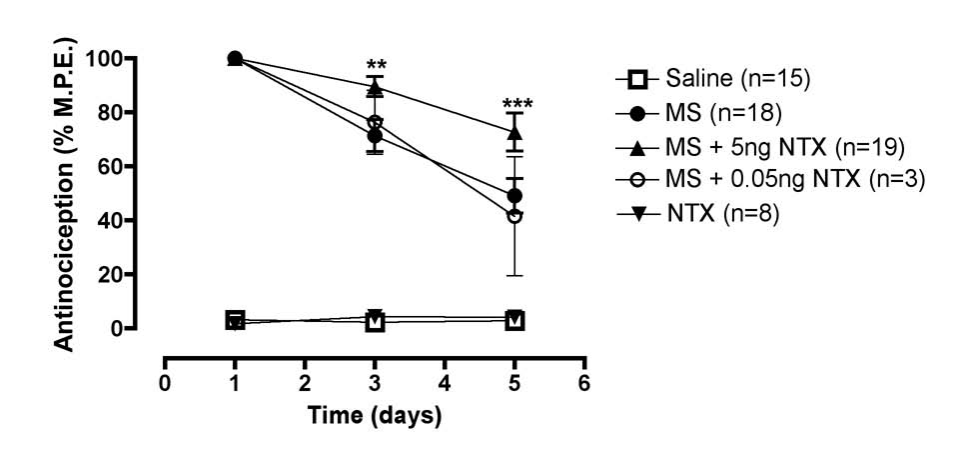
**Co-administration of ultra-low dose naltrexone (5 ng; NTX) with morphine (15 μg; MS) attenuated the loss in antinociception produced by morphine treatment alone in rats**. The lower dose, 0.05 ng NTX did not attenuate the loss in antinociception produced by chronic morphine. Ultra-low dose naltrexone alone did not produce significant antinociception as compared to vehicle (saline) treated controls. Statistical analyses were performed using a two-way ANOVA followed by Bonferroni *post hoc *test. The asterisk denotes a significant difference from morphine-treated rats. ** = p < 0.01, *** = p < 0.001.

Animals chronically administered intrathecal morphine (15 μg; MS) by lumbar puncture displayed a loss in antinociception on day 5 (49.1% maximum possible effect; MPE) as compared to day 1 treatment (100% MPE; Figure 1). Attenuation of the loss in antinociception was observed in animals that were co-administered ultra-low dose naltrexone (5 ng; NTX) with morphine, maintaining 72.7% of the maximum possible antinociceptive effect (Figure 1). This effect is dose dependent, as animals co-administered 0.05 ng naltrexone with morphine showed a loss in antinociception similar to animals treated with morphine only, maintaining 41.6% of the maximum possible antinociceptive effect on day 5. Control animals administered vehicle (saline) or ultra-low dose naltrexone alone did not produce changes in thermal nociceptive thresholds compared to baseline values (Figure 1).

### Ultra-low dose naltrexone attenuated morphine-induced increases in the expression of glial fibrillary acidic protein (GFAP) and CD3/CD11B (OX42)

**Figure 2:**
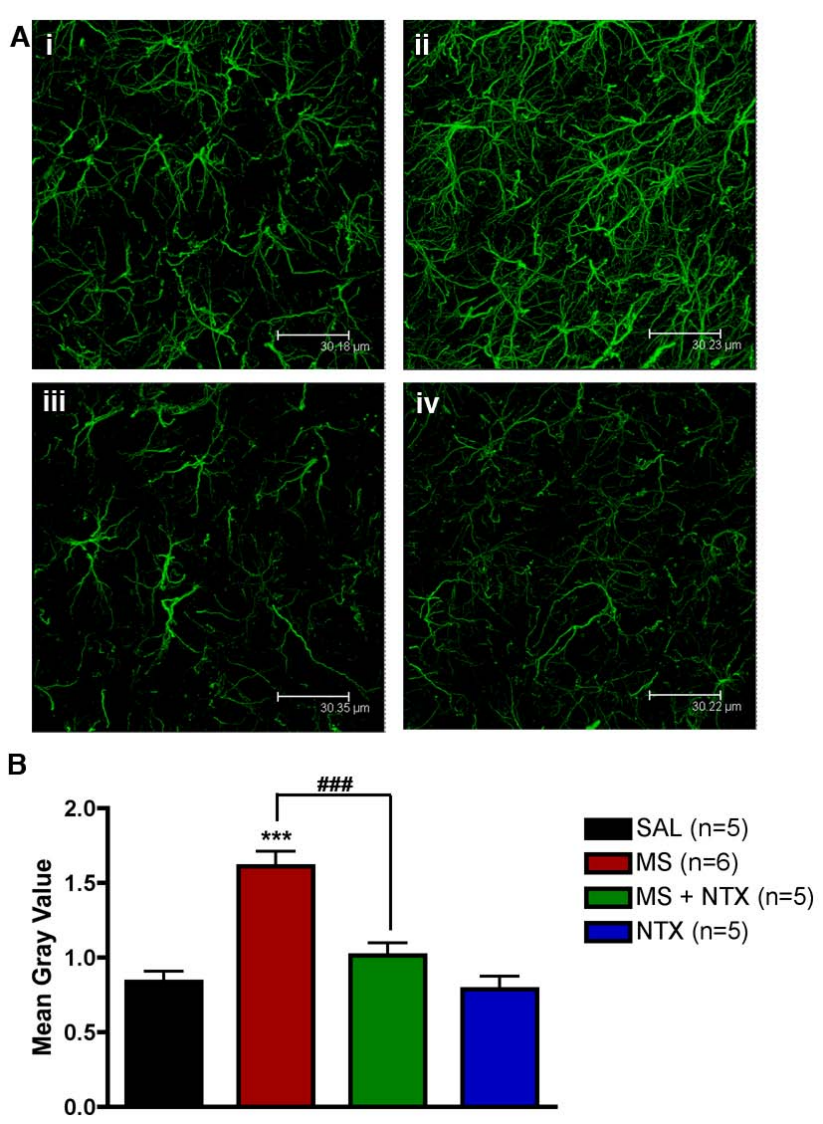
**Intensity of astrocyte labelling in the dorsal lumbar spinal cord of rats**. (A) Representative photomicrographs acquired by confocal microscopy of spinal cord sections labelled for the astrocytic protein, glial fibrillary acidic protein (GFAP). Spinal cord sections were collected from rats receiving intrathecal vehicle (saline; SAL) (i), morphine (15 μg; MS) (ii), morphine and naltrexone (5 ng; MS+NTX) (iii), or naltrexone (NTX) alone (iv). Photomicrographs were converted to gray scale and then analyzed to obtain mean gray values. Morphine treatment produced a significant increase in the amount of GFAP labelling as compared with saline-treated control. Attenuation of increased GFAP immuno-labelling was observed in animals co-administered ultra-low dose naltrexone with morphine (^### ^= p < 0.001 compared to morphine treatment). Naltrexone alone had no significant effect on GFAP immuno-labelling compared to saline control (p > 0.05). Data represent means ± s.e.m. for n = 6-8 sections per rat from n = 5-6 per group. Statistical analyses were performed by a one-way ANOVA followed by Tukey's *post-hoc *multiple comparison test. The asterisk denotes significant difference from saline-treated rats, *** = p < 0.001.

**Figure 3:**
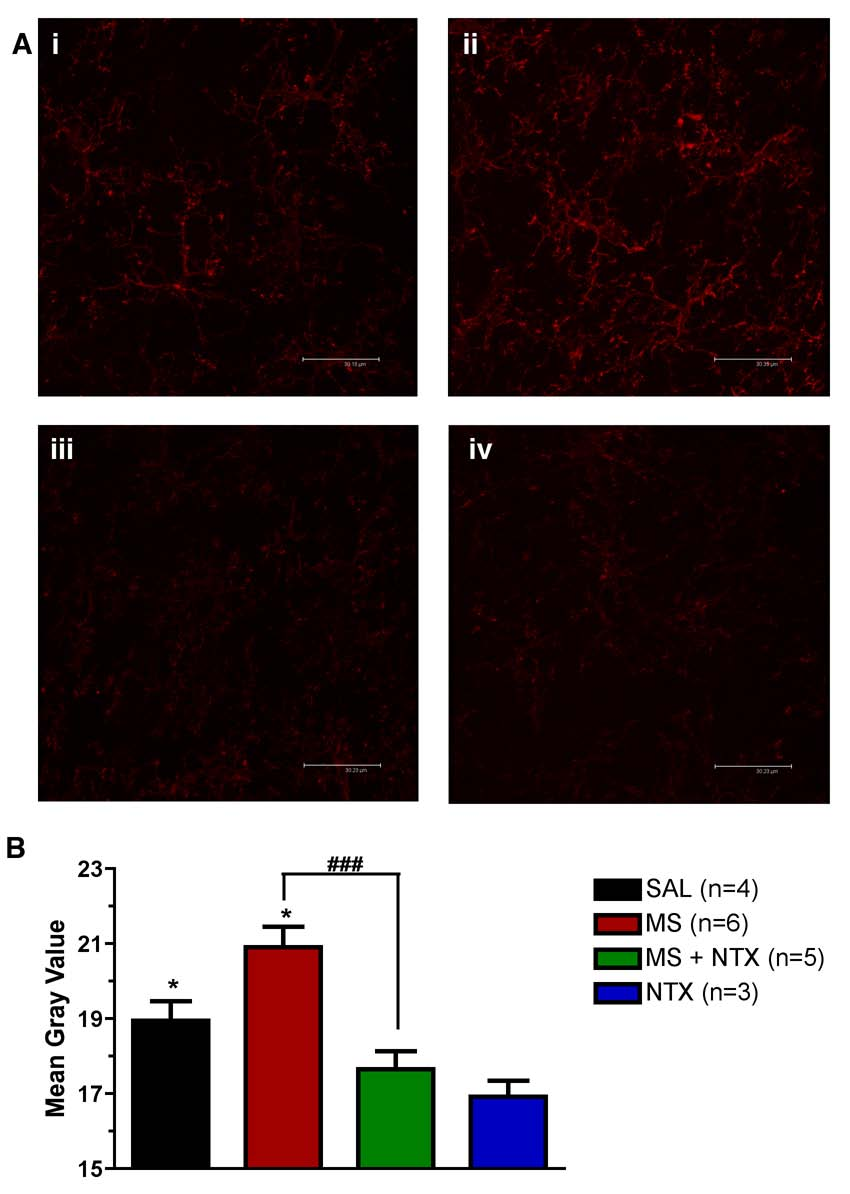
**Intensity of microglial labelling in the dorsal lumbar spinal cord of rats**. (A) Representative photomicrographs acquired by confocal microscopy of spinal cord sections labelled for the microglial marker, CD3/CD11B (OX42). Spinal cord sections were collected from rats receiving intrathecal vehicle (saline; SAL) (i), morphine (15 μg; MS) (ii), morphine and naltrexone (5 ng; MS+NTX) (iii), or naltrexone (NTX) alone (iv). Photomicrographs were converted to gray scale and then analyzed to obtain mean gray values. Morphine treatment produced a significant increase in the amount of OX42 labelling as compared with saline control. Attenuation of increased OX42 immuno-labelling was observed in animals co-administered ultra-low dose naltrexone with morphine (^### ^= *P *< 0.001 compared to morphine treatment). Naltrexone alone had no significant effect on OX42 immuno-labelling compared to saline control (*P *> 0.05). Data represent means ± s.e.m. for n = 6-8 sections per rat from n = 3-6 per group. Statistical analyses were performed by a one-way ANOVA followed by Tukey's *post-hoc *multiple comparison test. The asterisk denotes significant difference from saline-treated rats, * = *P *< 0.05.

Spinal cords collected from animals administered vehicle (saline), morphine (15 μg), combined morphine and ultra-low dose naltrexone (5 ng), or naltrexone alone were processed for immunohistochemical labelling. Representative images (Figure 2A i-iv) illustrate the increase in GFAP labelling observed in animals chronically administered morphine alone (Figure 2A ii) as compared to all other treatments. Quantification of GFAP labelling intensity (Figure 2B) revealed a significant increase in GFAP labelling in morphine only treated spinal cord sections as compared to saline controls. Co-administration of ultra-low dose naltrexone in combination with morphine did not produce a significant increase in GFAP expression as compared to saline-treated and naltrexone-treated controls (*P *> 0.05). No difference in GFAP expression was observed between saline-treated and naltrexone-treated treated sections. Similarly, increased CD3/CD11B expression was observed in morphine-treated animals compared to controls (Figure 3B), which was attenuated by co-administration of ultra-low dose naltrexone. Therefore, ultra-low dose naltrexone significantly attenuated the increase in GFAP and CD3/CD11B expression induced by chronic morphine administration.

### Ultra-low dose naltrexone attenuated morphine-induced astrocyte hypertrophy

**Figure 4:**
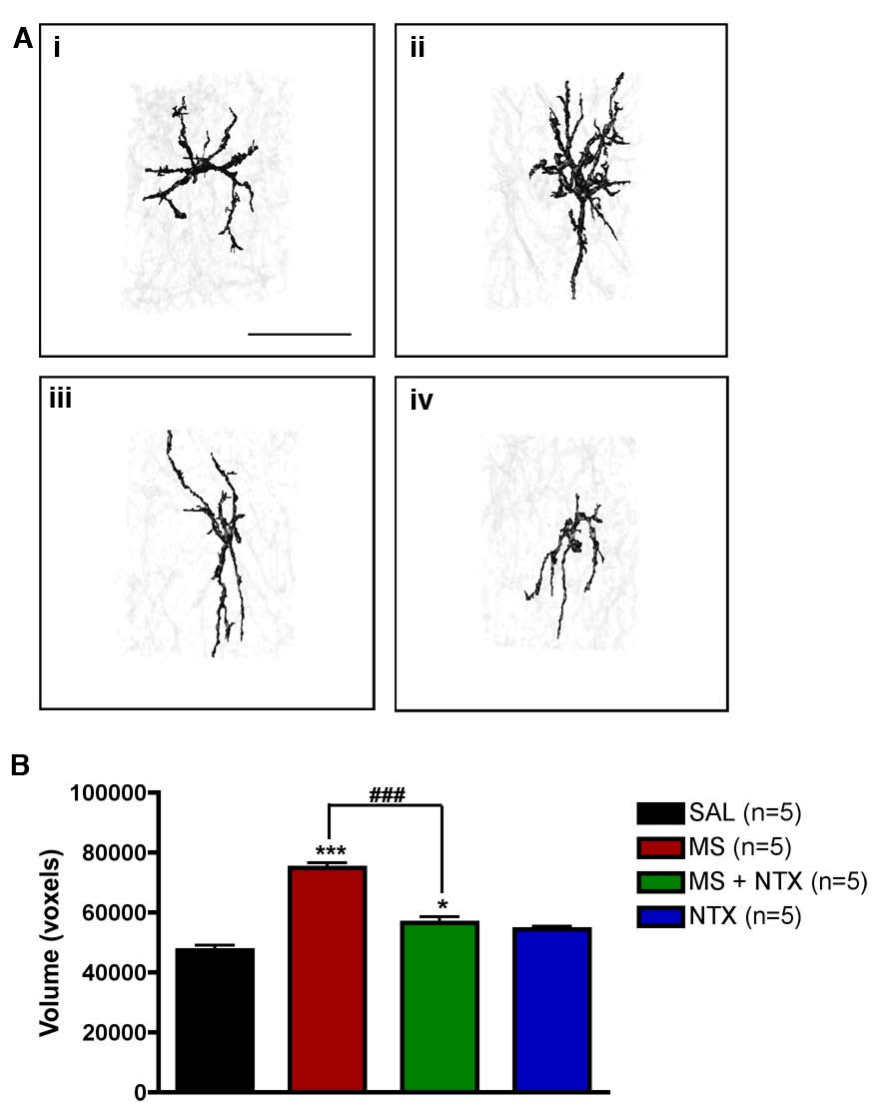
**Ultra-low dose naltrexone attenuates morphine-induced astrocyte hypertrophy**. (A) Representative three dimensional images of astrocytes from rats receiving intrathecal vehicle (saline, SAL) (i), morphine (15 μg, MS) (ii), morphine and naltrexone (5 ng, MS+NTX) (iii), or naltrexone (NTX) alone (iv). Morphine treatment produced a significant increase in astrocytic cell volume as compared with saline-treated and naltrexone-treated controls. Co-administration of ultra-low dose naltrexone with morphine attenuated this hypertrophy; astrocytes have significantly smaller volumes as compared to morphine only treatment (^### ^= p < 0.001). Data represent means ± s.e.m. for n = 12-24 cells per rat from n = 5 per group. Statistical analyses were performed by a one-way ANOVA followed by Tukey's *post-hoc *multiple comparison test. The asterisk denotes significant difference from saline-treated rats. * = p < 0.05, *** = p < 0.001. Scale bar, 30 μm.

Astrocyte cells were reconstructed in three dimensions from spinal cord sections obtained from rats chronically administered drug treatments. Representative images of astrocytes reconstructed from morphine-treated animals (Figure 4A i-iv) demonstrate hypertrophy characteristic of astrogliosis. Measurement of astrocyte cell volume confirmed that chronic morphine treatment produced significantly larger volumes compared to saline-treated controls (Figure 4B). Co-administration of ultra-low dose naltrexone with morphine attenuated this increase in cell volume (p < 0.001 compared to morphine-treated), however, astrocytes were still of significantly larger volumes than saline-treated controls but did not differ from naltrexone only controls. Moreover, naltrexone treatment alone did not significantly affect astrocyte volume compared to saline-treated controls. Thus, co-administration of ultra-low dose naltrexone significantly attenuated morphine-induced hypertrophy of astrocytes.

### Chronic morphine does not induce cell proliferation

**Figure 5:**
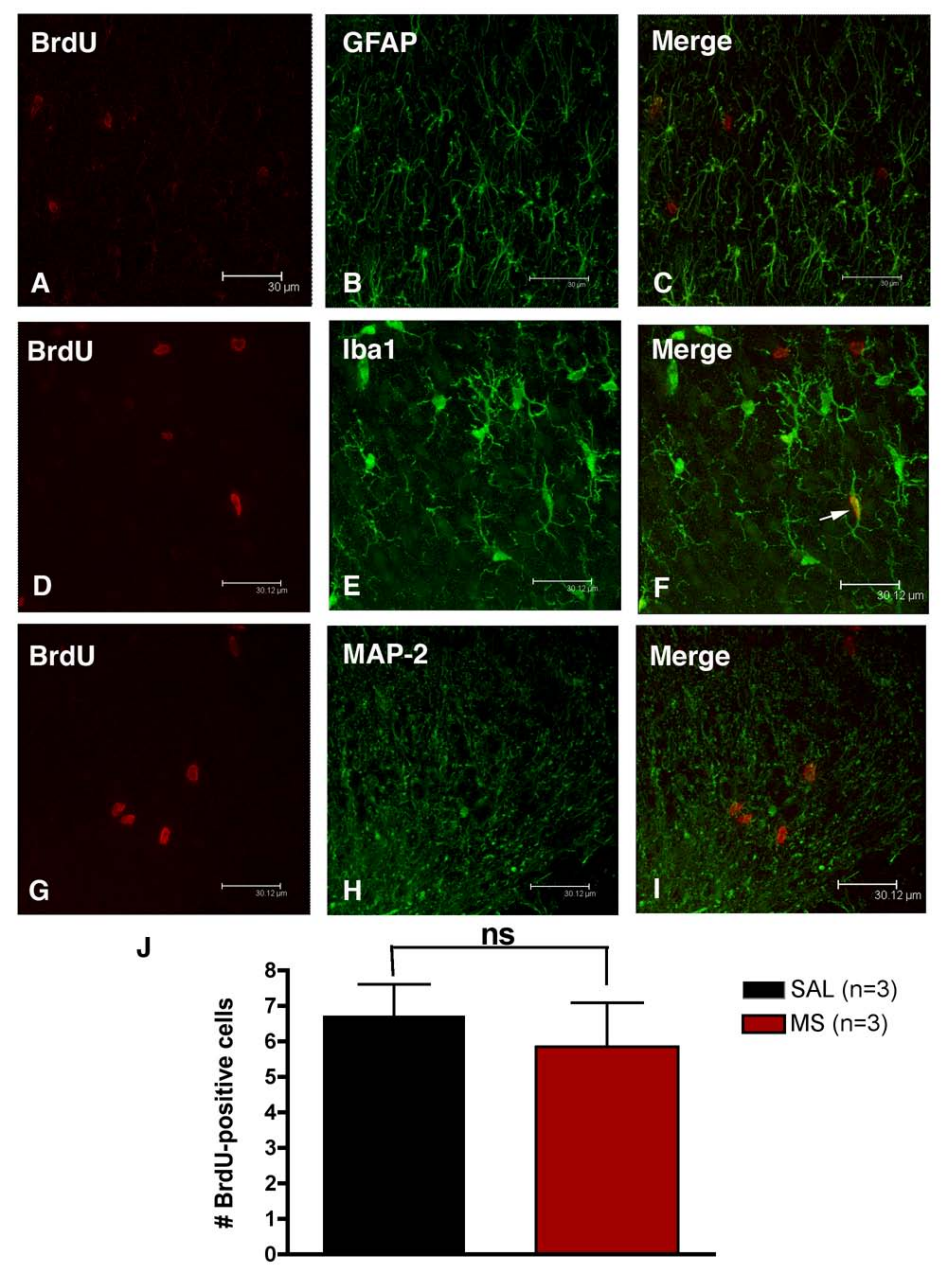
**The morphine-induced increase in astrocyte and microglial immuno-labelling is caused by cell hypertrophy, not proliferation**. Lumbar spinal cord sections were collected from animals administered BrdU (100 mg/kg) by intraperitoneal injection on days 1, 3, 5, and either intrathecal vehicle (saline; SAL) or morphine (15 μg; MS) once daily for five days by lumbar puncture. Representative photomicrographs acquired by confocal microscopy of spinal cord sections double labelled with 5-bromo-2-deoxyuridine (BrdU) and the astrocytic protein GFAP (B, C), the microglial marker Iba1 (E, F) or the neuronal marker MAP2 (H, I). No co-localization of BrdU-positive cells with GFAP or MAP-2-positive cells was observed. However, BrdU co-localized with a small number of Iba1 positive cells (arrow), suggesting a small portion of the newly formed cells were microglia or macrophages. (J). No difference was observed in the number of BrdU-positive cells in the dorsal horn (lamina II-IV) of lumbar spinal cord sections from animals administered chronic intrathecal saline or morphine. Data represent means ± s.e.m. for n = 6 sections per rat from n = 3 per group. Statistical analyses were performed by an un-paired t-test. ns = no significance. Scale bars, 30 μm.

The number of GFAP and OX42-positive cells present in lumbar spinal cord sections from animals chronically administered intrathecal vehicle (saline) or morphine (15 μg) were counted (Table 1). The number of GFAP-positive (astrocytes) and OX42-positive (microglia) cell bodies observed in spinal cord sections from morphine-treated rats was not significantly greater than the number in spinal cord sections from saline-treated controls. To confirm this finding, cell proliferation was assessed via 5-bromo-deoxyuridine (100 mg/kg, i.p; BrdU) experiments. BrdU was injected on alternative days 30 minutes prior to intrathecal administration of saline or morphine (15 μg) for 5 days. Immunohistochemical labelling of spinal cord sections collected from these animals revealed no significant increase in the number of BrdU-positive cells in morphine-treated animals compared to saline controls (Figure 5J). Double labelling of sections with the astrocytic marker GFAP (Figure 5A-C), or the neuronal marker MAP-2 (Figure 5G-I) revealed no co-localization with BrdU-positive cells. Iba1, the microglial and macrophage marker, co-localized with a portion of the BrdU-positive cells (Figure 5D-F). The results of the BrdU experiments confirm the cell counts of astrocytes and microglia, demonstrating that the chronic morphine treatment employed in this study does not induce spinal cord cell proliferation.

**Table 1:** Glial cell counts.

Cell Type	Saline	Morphine
Astrocyte (GFAP)	6.767 ± 0.502	8.295 ± 0.558

Microglia (OX42)	8.083 ± 0.911	8.886 ± 0.499

The number of astrocyte (GFAP-positive) or microglia (OX42-positive) cell bodies in the dorsal horn of spinal cord sections collected from rats receiving intrathecal morphine (15 μg, MS), or vehicle (saline). No significant difference was observed between treatment groups. Data represent means ± s.e.m. for n = 6-8 sections (150 μm × 150 μm) per rat from n = 4-6 per group. Statistical analyses were performed using an unpaired t-test.

## Discussion

The current study has provided additional evidence that ultra-low dose naltrexone attenuates the development of tolerance to the antinociceptive effects of morphine as previously demonstrated by Powell *et al *[20]. As the mechanism by which this phenomenon occurs is unknown, this study sought to investigate the contribution of glia in the actions of ultra-low dose opioid antagonists. Intrathecal catheterization has been shown to induce gliosis [25], therefore the current study used lumbar puncture drug delivery to reproduce the original behavioural findings of Powell *et al *[20]. Preliminary experiments investigated the effects of different ultra-low doses of naltrexone on morphine tolerance. In this study, the dose of naltrexone (0.05 ng) used in experiments by Powell *et al *[20] did not attenuate the loss in antinociception observed with chronic morphine administration. However, a hundred-fold greater dose (5 ng) preserved the analgesic effects of morphine throughout the treatment period, and thus was used to determine the effects on morphine-induced gliosis. In addition to the use of intrathecal catheters for drug delivery, another important difference in experimental protocol in the present study were the housing conditions; animals used in this study were housed in a room on a reverse light-dark cycle (lights off at 7:00 am), with all behavioural testing conducted during the animals' active (dark) phase. It is well accepted that pain responsiveness and endogenous opioids have circadian fluctuations in rats [26] and that morphine-induced antinociception is greater during the active phase compared to during the inactive light phase. These fluctuations may account for the greater dose of opioid antagonist required to attenuate tolerance in the present study compared to what has been previously published.

Current research has demonstrated that spinal glia are not merely support cells within the CNS as previously hypothesized (i.e. responsible for the maintenance of neurons and CNS homeostasis); they also actively communicate with neurons, are involved in the modulation of synaptic signalling and may be involved in the development of opioid tolerance. Chronic, but not acute, morphine administration, induces gliosis characterized by cell hypertrophy, and is associated with increased expression of GFAP [19,27-30] in astrocytes and CD3/CD11B (OX42) in microglia [31]. Reactive glial cells (microglia and astrocytes) can release a variety of pro-nociceptive and neuroexcitatory substances (e.g. prostaglandins, excitatory amino acids, interleukins, nitrogen oxide species, ATP, glutamate etc.), which may enhance pain transmission by nociceptive neurons [19,28,32-34].

This is the first report to demonstrate that co-administration of ultra-low dose naltrexone prevents morphine-induced gliosis, demonstrated by normalization of GFAP and CD3/CD11B expression and attenuation of increased astrocyte cell volume. The observed increases in GFAP/CD3/CD11B expression and astrocyte cell volume in spinal cord sections from animals chronically administered intrathecal morphine are consistent with gliosis and are in agreement with previous findings of astrocyte and microglial activation by chronic morphine administration [19,28-30]. As no significant difference was found in the number of immuno-positive cells or in the number of newly generated cells between morphine treated and saline controls, glial proliferation likely contributes very little to the observed increases in GFAP and CD3/CD11B expression. This finding is in agreement with that of Song and Zhao [19], in which chronic morphine resulted in increased astrocyte immunoreactivity with no difference in the number of cells from saline treated controls. In contrast, Narita *et al *[30] reported that astrocyte proliferation was induced by chronic morphine administration; however, no quantification of the number of GFAP-positive cells was reported. Agents that modify [19] or inhibit [18] activation of astrocytes and microglia prevent the development of morphine tolerance; thus inhibition of gliosis by ultra-low dose naltrexone may prevent the development of analgesic tolerance. This evidence, taken in concert with the findings of the current study, supports the hypothesis that spinal glia are involved in the development of morphine analgesic tolerance and in the mediation of nociception. It has also been reported that ultra-low dose naltrexone augments morphine antinociception in a model of pertussis toxin induced hyperalgesia [35].

While the present study provides strong support for the role of glia in the ultra-low dose effect, various molecular studies indicate that ultra-low dose antagonists may prevent opioid receptor coupling to stimulatory G-proteins (Gs). Classically, opioid activation of μ-opioid receptors results in coupling to inhibitory G-protein subunits (Gi/Go) and produces analgesia; however, following chronic opioid administration, increased coupling of μ-opioid receptors to Gs has been observed [21]. Therefore, increased excitatory stimulation via Gs-coupled μ-opioid receptors may oppose the analgesic effects mediated via Gi/Go signalling, and manifest as tolerance [21,36]. Wang *et al *[21] demonstrated that the switch in G-protein coupling to μ-opioid receptors induced by chronic morphine could be prevented by co-administering an ultra-low dose of naloxone, further supporting this hypothesis. Despite these advances, the switch in G-protein coupling to μ-opioid receptors induced by chronic morphine treatment has not been localized to a specific cell population within the spinal cord, and therefore, may occur in glia or nociceptive neurons. On the contrary, the effects of ultra-low dose antagonists may not be mediated by μ-opioid receptors but through a novel mechanism such as an interaction with filamin A [37] or Toll-like receptors [38]. Thus, future studies will aim to identify the mechanism by which ultra-low dose naltrexone alters gliosis.

## Conclusions

The results of this study may have a significant impact on the clinical management of moderate to severe pain. Patients currently treated with chronic opioid therapy may benefit not only from increased efficacy of combined opioid treatment [23,39], but may also experience fewer and less severe adverse effects [24,40], as sufficient analgesia can be achieved and maintained at lower opioid doses. Additionally, an understanding of the mechanism of action of opioid drugs will provide insight toward the development of more selective and efficacious pharmacological treatments for pain management. Not the least of which could be for improving treatment of chronic pain conditions such as neuropathic pain where glial activation is also evident, with reactive gliosis being a key contributor to the painful neuropathy [41-44]. Additionally, reduced opioid analgesic efficacy has also been reported in patients with neuropathic pain [45,46], however, co-administration of ultra-low dose antagonists with opioid agonists increased analgesic efficacy in animal models of neuropathic pain [47] and in clinical trials [23,24]. Future research will be required to determine if ultra-low dose naltrexone is able to alleviate established chronic pain.

## Methods

### Animals

Adult male Sprague-Dawley rats (180-200 g; Charles River, Québec, Canada), were housed in groups of two with *ad libitum *access to food and water, and maintained on a reverse 12/12 h light/dark cycle. All behavioural experiments were performed during the dark phase of the cycle, and animals were handled prior to experimentation in order to reduce stress-related analgesia. All experimental protocols were approved by the Queen's University Animal Care Committee, and complied with the policies and directives of the Canadian Council on Animal Care and the International Association for the Study of Pain.

### Drug treatments

Morphine was purchased from Sabex, Kingston General Hospital, Kingston, Ontario, Canada. Naltrexone and 5-bromo-2-deoxyuridine (BrdU) were purchased from Sigma (St. Louis, MO, USA). Animals were separated into one of five groups receiving i) morphine (15 μg; n = 18), ii) morphine and naltrexone (5 ng; n = 19), iii) morphine and naltrexone (0.05 ng; n = 3), iv) naltrexone (5 ng) alone (n = 8), or v) saline (n = 15). Intrathecal (i.t.) administration of all drugs (diluted in saline to 30 μl volume) was accomplished by way of lumbar puncture between the L4 and L5 vertebrae under brief isofluorane anesthesia. Successful drug placement was confirmed by a vigorous tail flick upon injection.

To determine if chronic morphine treatment induced cell proliferation, animals received 5-bromo-2-deoxyuridine (BrdU, 100 mg/kg; prepared in a concentration of 25 mg/ml in 0.007 N NaOH and saline), injected intraperitoneally (i.p.) on days 1, 3, and 5. Animals were separated into two groups receiving intrathecal morphine (15 μg/15 μl; n = 3) or saline (15 μl; n = 3) by lumbar puncture under brief isoflurane anaesthesia for 5 days. Saline or morphine was injected 30 minutes after BrdU injections.

### Behavioural tail flick assay

The effects of drug administration on thermal nociceptive responses were assessed on Days 1, 3 and 5 of the study using the tail flick assay. In brief, a beam of radiant light was applied to a spot marked 5 cm from the tip of the tail, and the latency to a vigorous tail flick was measured. Three baseline latencies were measured prior to drug injection to determine the normal nociceptive responses of the animals. A cut-off time of three times the animal's average baseline was imposed to avoid tissue damage in the event that the animal became unresponsive following drug injection. Rats were then injected intrathecally with their respective treatments, and the thermal latency measured at 30 minutes post-injection, as previous studies have found that the peak antinociceptive effects of morphine occur at this time point [48]. Tail-flick values were converted to a maximum possible effect (% MPE): (post-drug latency - baseline) ÷ (cut-off latency - baseline) × 100. Statistical analyses were performed using a two-way analysis of variance (ANOVA), followed by Bonferroni's *post-hoc *multiple comparisons test to determine between group differences. P values less than 0.05 were considered significant. All behavioural testing was performed by the experimenter blind to drug treatment.

### Immunohistochemistry

On day 6, 24 h after the last injection, rats (n = 3 per drug treatment) were deeply anesthetized with sodium pentobarbital (75 mg/kg, i.p.; MTC Pharmaceuticals, Cambridge, Ontario, Canada) and transaortically perfused with 4% paraformaldehyde (PFA) in 0.1 M phosphate buffer (PB; 500 ml, pH 7.4). The spinal cords were removed by spinal ejection and post-fixed in the above fixative for 1 hour on ice and cryoprotected in 30% sucrose in 0.1 M PB for 48 hours at 4°C. Lumbar segments were isolated and cut into 40 μm transverse sections on a freezing sledge microtome and collected in 0.1 M Tris buffered saline (TBS; pH 7.4).

Free-floating sections were incubated in a blocking solution containing 5% NGS in TBS-T (TBS and 0.2% Triton X-100), followed by incubation with a rabbit polyclonal antisera recognizing glial fibrillary acidic protein (GFAP; 1:2500 working dilution; DakoCytomation, Ontario, Canada) to label astrocytes and a mouse monoclonal antisera recognizing OX42 (1:1000 working dilution; Serote, NC, USA) to label CD3/CD11B receptors on microglia. Spinal cord sections were incubated overnight at 4°C with both primary antibodies, followed by incubation with goat anti-rabbit and goat anti-mouse secondary antibodies (1:200 working dilution; Molecular Probes, Invitrogen, Ontario, Canada) conjugated to Alexa 488 and Alexa 594 fluorophores, respectively. To assess non-specific labelling, control sections were processed in the absence of primary antibody. Sections were mounted on glass slides, air-dried and cover-slipped using Aquamount (Fisher Scientific, Ontario, Canada).

BrdU immuno-labelling was performed as described by Suter *et al *[49]. Briefly, spinal cord sections were heated in solution containing 50% formamide, 50% 2× saline sodium citrate (SSC) for 2 h at 65°C. Sections were further incubated at 37°C for 30 min in 2N HCL then placed in 0.1 M borate buffer (pH 8.5). Sections were incubated in blocking solution, followed by incubation with a mouse monoclonal antibody against BrdU (1:500, Chemicon, Temecula, CA). To identify the phenotype of newly formed cells, sections were double labelled with one of the following antibodies: rabbit anti-GFAP (for astrocytes, 1:2500), rabbit anti-Iba1 polyclonal antibody (ionizing calcium-binding adaptor molecule, for microglia and macrophages, 1:1000; Wako, Richmond, VA), or rabbit anti-MAP-2 polyclonal antibody (microtubule-associated protein 2, for neurons, 1:1000; Chemicon, Temecula, CA). Sections were then incubated with goat anti-rabbit and goat anti-mouse secondary antibodies (1:200) conjugated to Alexa 488 and Alexa 594 fluorophores, respectively, and mounted as described above.

Imaging of immunoreactive cells was performed as previously described [50]. In brief, immunoreactive cells were imaged using the Leica TCS SP2 multi photon confocal microscope (Leica Microsystems Inc, Ontario, Canada). Images were taken within the dorsal horn (lamina III-V) at 63× magnification for quantification of intensity. Serial images (twenty-five to thirty-five) were captured at 100× magnification, at 0.75 μm increments throughout the z plane in the deep and superficial dorsal horn (4 series per section, 3 sections per animal).

For quantification of the intensity of antibody labelling, images were converted to gray scale using Adobe Photoshop 7.0. Using Image J (NIH), the mean gray values were measured and the average within each treatment group calculated and expressed as mean ± s.e.m. For quantification of GFAP, OX42 and BrdU-positive cells, immunolabelled cell bodies were counted for each section (150 μm × 150 μm) and the average within each treatment group calculated and expressed as mean ± s.e.m. To quantify astrocyte volume, images taken at 100× magnification were stacked and reconstructed in three-dimensions using ImagePro Plus v5.0 software (MediaCybernetics, MD, USA). Total cell volume was calculated for each reconstructed cell. The average volume for cells within each treatment group was calculated and expressed as mean ± s.e.m. Mean intensities and 3D volumes were analyzed by one-way ANOVA followed by Tukey's post-hoc multiple comparison test. Differences in cell numbers were analyzed by unpaired T-tests. P values less than 0.05 were considered significant. All quantification data was collected by experimenter blind to drug treatment.

## Competing interests

The authors declare that they have no competing interests.

## Authors' contributions

TAM participated in the design of the study, carried out data collection, statistical analysis and interpretation, and drafted the manuscript. BM participated in the conception and design of the study, data interpretation, and editing of the manuscript. CMC participated in the conception and design of the study, data interpretation and editing of the manuscript. All authors read and approved the final manuscript.
